# Microencapsulation of Probiotics by Calcium Alginate-gelatinized Starch with Chitosan Coating and Evaluation of Survival in Simulated Human Gastro-intestinal Condition 

**Published:** 2014

**Authors:** Mohammad Ali Khosravi Zanjani, Babak Ghiassi Tarzi, Anousheh Sharifan, Nima Mohammadi

**Affiliations:** a College of Food Science and Technology, Science and Research Branch, Islamic Azad University, Tehran, Iran.; b Young Researchers and Elites Club, Science and Research Branch, Islamic Azad University, Tehran, Iran.

**Keywords:** Microencapsulation, Calcium alginate-gelatinized starch, Chitosan, Inulin, Simulated gastro-intestinal condition

## Abstract

Microencapsulation as one of the most modern methods has considerable effects on probiotic survival. In this study *Lactobacillus casei *(ATCC 39392) and *Bifidobacterium bifidum *(ATCC 29521) were encapsulated using calcium alginate-gelatinized starch, chitosan coating and inulin via emulsion technique, and were incubated in simulated gastric juice (along with pepsin, pH=1.5) and simulated intestinal juice (along with pancreatin and bile salts, pH = 8) for 2 hours at 37 ^o^C. The morphology and size of microcapsules were measured by scanning electron and optical microscopy. The results indicated that the survival of microencapsulated probiotic increased significantly in simulated gastro-intestinal condition (P < 0.05). Chitosan coating played a significant role in the protection of probiotic bacteria in simulated gastro-intestinal condition and the diameter of the microcapsules increased with the addition of chitosan coating. In general, this study indicated that microencapsulation with alginate-gelatinized starch coated with chitosan could successfully and significantly protect probiotic bacteria against adverse condition of simulated human gastro-intestinal condition.

## Introduction

Probiotics are defined as live microorganisms which, when administrated in adequate amounts confer health benefit on humans (1). Bacteria belonging to genera *Bifidobacterium *and *Lactobacillus *are often used as probiotic supplements (2). Some of these health advantages are: alleviation of symptoms of lactose malabsorption, cancer suppression, resistance to infectious gastro-intestinal disease, and improving digestion (3-5). The daily intake of probiotic bacteria should exceed 10^6 ^living bacteria per milliliter or per gram of the product (4). 

The prebiotics are non-digestible food ingredients that affect the host by selectively stimulating the growth and activity of bacteria in the colon (6). The most frequently studied examples are inulin and FOS (fructooligosaccharides) are known to have a positive effect on human health and promote the survival of probiotic bacteria (6-9). 

Microencapsulation with hydrocolloids as one of the most modern methods has remarkable effects on probiotic survival (3). Encapsulation process is a promising technique for probiotics protection against adverse conditions to which probiotics can be exposed (3, 10-13). Several studies have been carried out investigating the protective role of this technique. Microencapsulation can be employed via extrusion and emulsion techniques. Emulsion technique is a method for encapsulation of probiotic bacteria in the capsules smaller than 1 mm (10). Carbohydrate polymers such as alginate have been used in various microencapsulation procedures (2, 14). Alginate is a natural heteropolysaccharide composed of D-mannuronic and L-guluronic acid residues joined linearly by (1-4) glycosides linkages (2, 10). It is simple to handle, non toxic, low cost, ease of operation, and mild processing conditions (10, 11). Different studies have shown that the addition of starch as a filler material in the alginate capsule matrix improved the viability of probiotic cultures (2, 11). Gelatinized starch creates an integrated and uniform mixture with alginate because of its good binding capacity and sufficient solubility (compared to non-gelatinized starch) and this in turn, facilities capsule formation and efficiency in microencapsulation procedure (11, 12). However, alginate microcapsules (calcium alginate) are chemically susceptible to disintegration in the presence of excess monovalent ions, Ca^2+^-chelating agents such as phosphate and citrate and harsh chemical conditions such as those of low pH (13-15). To increase the stability of alginate microcapsules and to decrease the loss of encapsulated material, the alginate microcapsules are coated with polycationic polymers such as chitosan and poly-L-lysine (15, 16). Chitosan is a natural polysaccharide comprising glucoamine and N-acetylglucoamine with unique polycation characteristics (14, 17). The polycationic nature of chitosan leads to a strong interaction of carboxylic groups of alginate with the amine groups of chitosan results in the formation of a membrane (14). Because of the high protection of chitosan for viable cells, it has been used as a coating agent for probiotic microencapsulation (13). There are many advantages of the chitosan coating, such as non toxicity, good biocompatibility and the improvement of drug bioadhesive and payload properties (19, 20).

Many results have been reported concerning the use of chitosan-coated microcapsules as drug delivery formulation (17-23). 

Different studies have shown that calcium alginate microcapsules are better protected in the presence of coating polymers and prebiotics such as resistant starch, with the increase in survival of bacteria, under different conditions than when bacteria were tested in the free state (11, 24-28). Although some studies have reported that the presence of different hydrocolloids in the gastro-intestinal medium significantly improves survival of probiotic cells, so far no studies have been carried out using the technique of microencapsulation by calcium alginate-gelatinized starch with chitosan coating in order to verify the possibility of increasing the viability of *B. bifidum *ATCC 29521 and *L. casei *ATCC 39392 towards simulated human gastro-intestinal condition (along with pepsin and pancreatin). Furthermore, this study was undertaken on the alginate encapsulation of probiotic bacteria with inulin as a prebiotic compound. The purpose of this study is to enhance alginate microencapsulation by chitosan-coating and to evaluate their ability to improve the survival of *B. bifidum *and *L. casei *during exposure to simulated human gastro-intestinal condition. Also this study assesses the influence of coating materials on size and morphology of microcapsules by optical and scanning electron microscopy. 

## Experimental

*Preparation of cell suspension*

*Lactobacillus casei *ATCC 39392 (American Type Collection Culture) and *Bifidobacterium bifidum *ATCC 29521 were purchased from Iran Scientific and Industrial Organization. Lyophilized cells were inoculated in MRS broth (de Man-Rogasa-Sharpe) for 24 h under aerobic and anaerobic conditions at 37 °C, respectively and biomasses were then harvested by centrifuging at 4000 rpm for 10 min at 4 °C. The cultures were then washed twice by sterile saline solution (0.9%) and used in the microencapsulation process (10).

*Microencapsulation procedure*

All glassware and solutions used in the protocols were sterilized at 121 °C for 15 min. The encapsulation way for making microcapsules was a modified version of methods basically reported by Donthidi *et al*. (11) in 2010 and Sultana *et al*. (25) in 2000. Briefly, 2 g of maize starch (Sigma-Aldrich S4126) was added to 100 mL distilled water and boiled until it formed a gel, then sodium alginate (Sigma-Aldrich 71238) and required concentrations of inulin (1 %) were added and stirred until they were dissolved or dispersed. Then probiotic cultures of each bacteria were transferred to the carrier solutions with stirring under sterile conditions to ensure uniform distribution of the cells. The final mixture was suspended in 500 mL vegetable oil containing 0.2% tween 80 and mix (350 rpm for 20 min, Heydolph Stirrer, Germany) until they appeared creamy. Capsules were prepared by adding 200 mL calcium chloride 0.1 M into a mixture, the phase separation of oil/water emulsion occurred. The mixture was allowed to stand for 30 min to settle calcium alginate capsules in the bottom of beaker at the calcium chloride layer (Water phase). The oil layer was drained and capsules in calcium chloride solution were harvested by low speed centrifuge at 350 g for 10 min and kept in 0.1% peptone solution at 4 °C. 

*Coating with chitosan*

Chitosan aqueous solution was prepared according to Krasaekoopt *et al. *(13) in 2004. In brief, chitosan (Low molecular weight, Sigma-Aldrich 448869) was dissolved in 90 mL distilled water acidified with glacial acetic acid to achieve a final chitosan concentration of 0.4% (w/v). The pH was then adjusted to between 5.7 and 6 by adding 1 m NaOH. The mixture was filtered through filter paper (*Whatman NO. 41) *and autoclaving at 121 °C for 15 min. Then 20 g of washed microcapsules (alginate-gelatinized starch) were immersed in 100 ml of chitosan solution and shaken at100 rpm for 40 min on an orbital shaker for coating. The chitosan-coated microcapsule were washed with peptone solution, and kept in 0.1% peptone solution at 4 °C.

*Preparation of simulated gastric and intestinal juices and inoculation of cells*

The simulated juices were prepared according to Brinques *et al*. (27) in 2011 and Michida *et al*. (29) in 2006. Simulated gastric juices were prepared by dissolving pepsin (Sigma-Aldrich P7000) in sterile sodium chloride solution (0.5%, w/v) to a final concentration of 3.0 g/L and adjusting the pH to 1.5 with hydrochloric acid. Simulated intestinal juices were prepared by suspending pancreatin (Sigma-Aldrich P-1500) in sterile sodium chloride solution (0.5%, w/v) to a final concentration of 1 g/L, with 4.5% bile salts (Oxoid, Basingstoke, UK) and adjusting the pH to 8.0 with sterile NaOH (0.1 M). Both solutions were filtered for sterilization through a 0.22 μm membrane. The probiotic bacteria *L. casei *and *B. bifidum *were inoculated to the simulated gastro-intestinal juice individually in three different forms, non-encapsulated, encapsulated with calcium alginate-gelatinized starch, and calcium alginate-gelatinized starch coated with chitosan. It should be noted that inulin exists merely in the last two forms as a prebiotic compound. Then one gram of freshly encapsulated bacteria samples or 1 mL of cell suspensions (free cells) were gently mixed with 10 mL of sterile simulated gastric juice or sterile simulated intestinal juice and incubated at 37 °C for 30, 60, 90, and 120 min. Surviving bacteria were numerated by pour plate counts in MRS agar aerobically incubated at 37 °C for *L. casei *and in MRS agar anaerobically incubated at 39 °C for *B. bifidum *for 2 days. 

*Size and morphology of microcapsules*

The mean diameter of beads was measured by optical microscopy (Master sizer Malvern 2000 UK). The diameters of 100 randomly selected capsules were measured by using measurement software (Leica Qwin 550). The morphology of the capsules was observed using scanning electron microscope (LEO 440 I, England). The capsules were placed on a specimen aluminum stub with the help of double sided sticky tape and were coated by sputter coater for 2 min at an accelerating voltage of 15 kV (10).

*Release of entrapped bacteria*

The capsules containing probiotic bacteria were released by citrate buffer (pH= 6.0, 1 %) reported by Mokarram *et al*. (10) in 2009. One gram of capsules was transferred to 9 mL buffer. The solution was stirred on a shaker for 15 min vigorously (IKA-MS2, Minishaker, USA) until bacteria released from capsules completely. The counts (CFU/g) were determined by plating on MRS agar plates and incubating for 48 h at 37 °C. The free bacteria were treated similarly. All samples were counted in triplicates. The viability of microencapsulated probiotics in sterile sodium chloride solution (0.5%, w/v) and peptone water was also measured at 4 °C for three months. The microcapsules were dissolved in the appropriate buffer solution after three months and they were used to determine the total number of viable cells.

*Encapsulation yield*

Encapsulation yield (EY) *i.e*. the number of bacterial cells that survived the process and encapsulated inside the microcapsules was calculated as follows:

*EY *= (N/N_0_) × 100

Where N_0_ is the number of viable bacteria in CFU/mL of culture and N is the number of viable bacteria in CFU/g of microcapsules.

*Statistical analysis*

A complete randomized factorial design was used for all analysis and all results were means of three replicates. Data analysis was carried out using Statistical Package for Social Sciences (SPSS) Inc. software (20: SPSS Inc., Chicago, IL). The mean differences were analyzed by Duncan’s multiple range test.

## Results and Discussion

*Shape and size of microcapsules and Encapsulation yield*

The size and encapsulation yield of different microcapsules are presented in Table 1. There were no significant differences (P > 0.05) between coated and uncoated beads in microencapsulation yield. The results also represented that due to the gentle methods used, there was no significant (P > 0.05) loss of viability for *Lactobacillus casei *and *Bifidobacterium bifidum *during microencapsulation procedure and probiotics were successfully entrapped (Table 1). Our findings indicated that the average yield of all samples after encapsulation and coating was 97.4 %. These findings are in agreement with the results of Mokarram *et al*. (10) in 2009 who indicated that the mean encapsulation yield was 99.8 % in emulsion technique. Furthermore the viability of probiotics in sterile sodium chloride solution (0.5%, w/v) and peptone water at 4 °C showed that the number of bacteria in all the samples (microencapsulated forms) remained significantly unchanged. 

**Table 1:** Size and encapsulation yields of different microcapsules

**Probiotic**	**Microcapsule type**	**Size of microcapsules (μm)**	**Microencapsulation yield (%)**
*Lactobacillus casei*	Alginate-gelatinized starch	90 ± 1.69^(a)^*	98.12%
	Chitosan coated	123 ± 2.11^(b)^	97.21%
*Bifidobacterium bifidum*	Alginate-gelatinized starch	94 ± 1.72^(a)^	97.85%
	Chitosan coated	125 ± 1.81^(b)^	96.42%

* Means with different letter in a column are significantly different (P<0.05).

**Figure 1:**
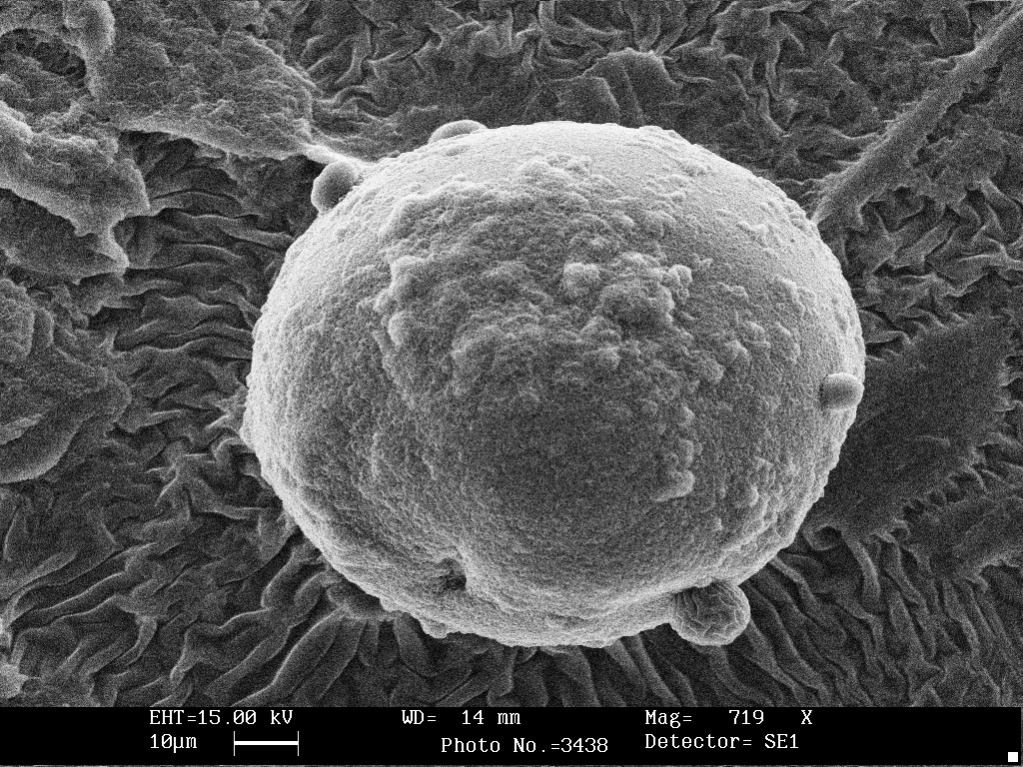
Scanning electron photomicrograph showing calcium alginate-gelatinized starch without chitosan coating containing *Lactobacillus casei.*

**Figure 2:**
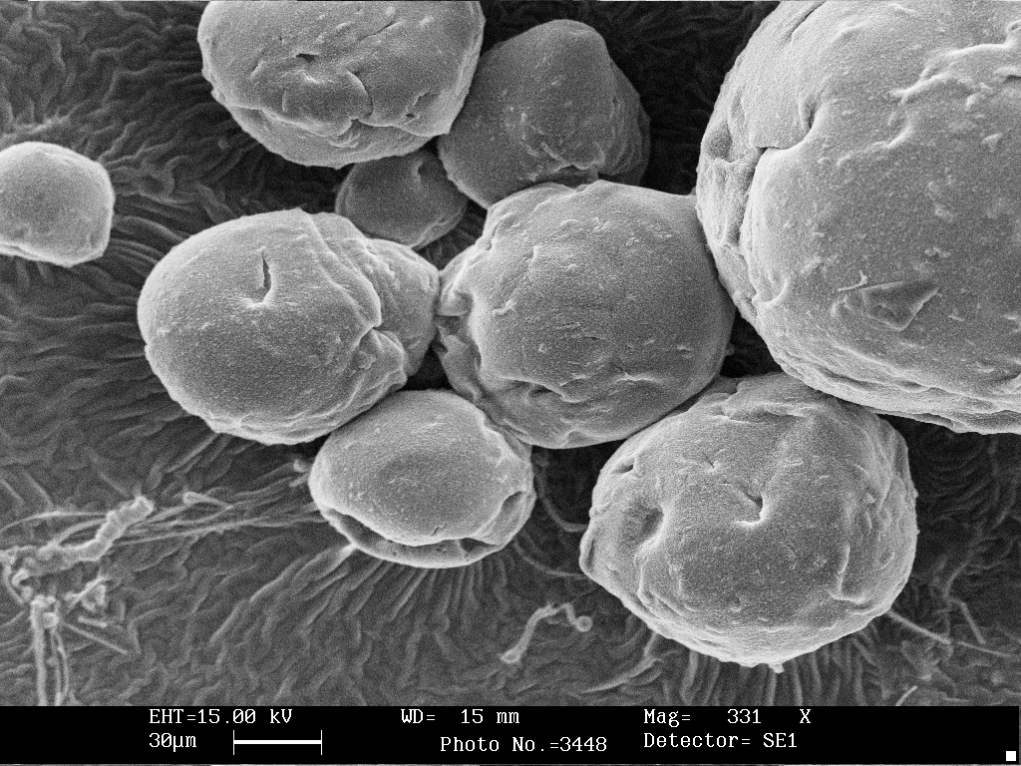
Scanning electron photomicrograph showing calcium alginate-gelatinized starch coated with chitosan containing *Bifidobacterium bifidum.*

Scanning electron microscopy showed that the shape of all microcapsules was generally spherical and uniform and starch granules were present on the surface of the capsules without coating (Figure 1). Chitosan coating changed morphology and shape of microcapsules and modified the surface of alginate beads (Figure 2). The size of calcium alginate-starch was analyzed by measurement software (Leica Qwin 550). The mean diameter of microcapsules without chitosan coating was 92 ± 1.709 μm. The size of the alginate capsules increased with the addition of chitosan coating. The diameter of chitosan-coated microcapsules was 124 ± 1.96 μm, which was significantly higher than that of uncoated microcapsules (Table 1). In this technique, the capsules are formed in micron range size. Furthermore, several reports have shown that capsules with micron range size delivered soft texture when they are added to food product (18, 24, 25, 28). Microcapsules with double coating sodium alginate used in Mokarram *et al*. (10) study in 2009 were also micron size range (75.339 ± 0.209 μm). Krasaekoopt *et al*. (13) in 2004 obtained a larger chitosan coated capsule size of 1.89 mm via extrusion technique. Moreover, Koo *et al*. (16) in 2001 reported that the shape and size of the beads were not changed when chitosan was added to alginate beads. Different studies have shown that size reduction of the capsules to less than 100 μm would not offer any significant increase in survival rate of the probiotics on the gastric secretion condition (18, 30). 

*Survival of free and microencapsulated probiotics in simulated gastric juice*

**Figure 3:**
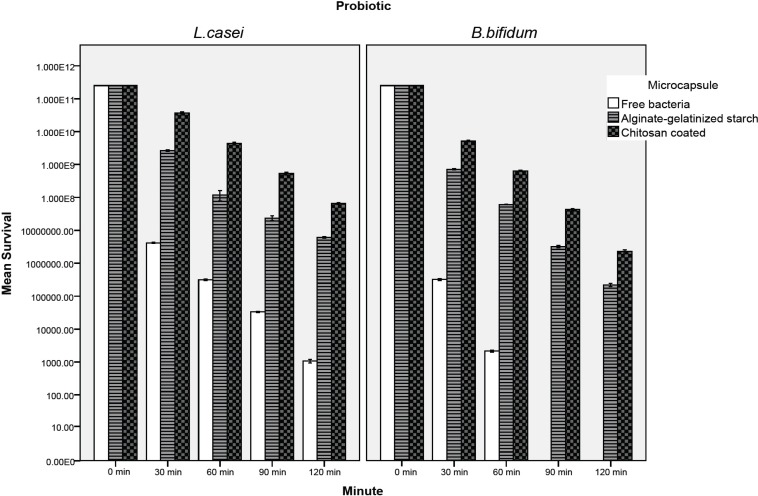
Survival of free and microencapsulated *lactobacillus casei *and *Bifidobacterium bifidum *in simulated gastric juice

**Figure 4:**
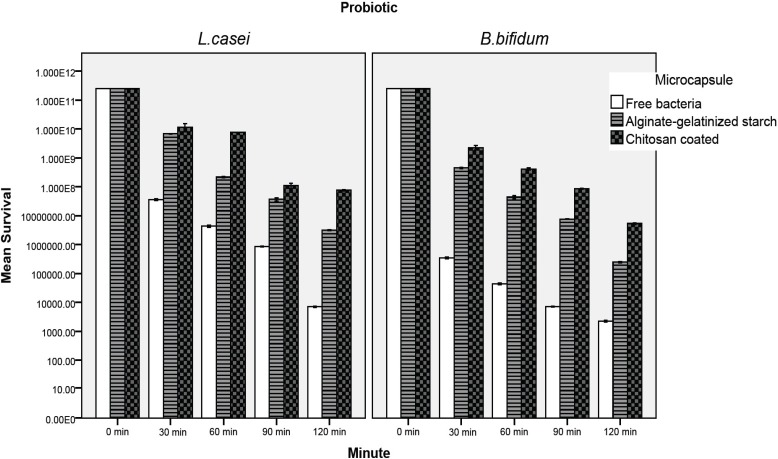
Survival of free and microencapsulated *lactobacillus casei *and *Bifidobacterium bifidum *in simulated intestinal juice

Figures 3 and 4 show the viability of free and encapsulated probiotic bacteria during 120 min of incubation in the simulated gastro-intestinal condition. Figure 3 illustrates that the survival of probiotics was lower in gastric juice and decreased further as the incubation period increased. After 120 min, the survival of free *L. casei *decreased from 2.51×10^11^ to 1.1×10^3^ CFU/ mL. However, the cell number of coated and uncoated microencapsulated *L. casei *decreased to 6.3×10^7^ and 6.2×10^6^ CFU/mL respectively after 120 min. In the case of *L. casei, *the survival of cells in both coated and uncoated capsules was significantly (P < 0.05) better than that of free cells and alginate-gelatinized starch with chitosan coating provided the best protection for both probiotics (Figure 3). Exposure to simulated gastric juice resulted in a considerable decrease in the total number of free *bifidobacterium bifidum*. Due to its low acid resistance there was no survival of free *B. bifidum *in the presence of simulated gastric juice. The free *B. bifidum *cell numbers decreased to an undetectable level within 90 min (Figure 3). This finding is in agreement with those of Lee *et al*. (15) in 2004, and the similar study done by Krasaekoopt *et al*. (13) in 2004 who reported that no *Bifidobacterium bifidum *ATCC 1994 survived in the simulated gastric pepsin free-environment of pH 1.55 for 15 min. Chávarri *et al. *(9) in 2010 reported that encapsulation in chitosan-coated alginate microspheres significantly improved the survival of *Lactobacillus gasseri *and *Bifidobacterium bifidum *in simulated gastric juice along with pepsin. Many scientists have also reported that the survival rate of bifidobacteria in alginate microcapsules was higher than that of free cells (30-32). However, Sultana *et al*. (25) in 2000 found that encapsulation of bacteria in alginate beads did not effectively protect the probiotics from high acidity. Many studies have shown coating the alginate matrix could increase the survival of bacteria due to curbing the diffusion of calcium ions outside of capsules (9, 10, 13). Mokarram *et al*. (10) in 2009 showed that *L. acidophilus *and *L. rhamnosus *exposed to simulated gastric juice without pepsin had higher viability when encapsulated in calcium alginate with double coating sodium alginate. They indicated that the reduction of pore size and distribution of gastric juice in double layer membrane lead to limitation of interaction between cells with the gastric juice. According to our study coated microcapsules provide the best protection in simulated gastric juice. Since gelatinized starch with chitosan coating reduces the porosity of alginate beads and decreases the leakage of the encapsulated probiotic, this structure can demonstrate more resistance in harsh conditions such as acidic pH of simulated gastric juice. Furthermore the increase in viable counts of bacteria could be attributed to the addition of inulin, which acts as a prebiotic. Alginate and prebiotics such as inulin or oligosaccharides tend to be synergistic in gelling and as a result may help maintain and improve the degree of protection to bacterial cells (6, 7, 34). Sultana *et al*. (25) in 2000 showed that the addition of resistant maize starch (non gelatinized) as a prebiotic to an alginate matrix increased the recovery of encapsulated cells; however, they remained sensitive to simulated gastric juice. Capela *et al. *(6) in 2006 reported that prebiotis such as Hi-maize, FOS and inulin were helpful in improving viability of probiotic organisms in fresh yoghurt during storage. They found that the improved viability in fresh yoghurt is possibly due to prebiotics providing extra solids, which tend to protect cells from injury. 

*Survival of free and microencapsulated bacteria in simulated intestinal juice*

The effect of the simulated intestinal juice on the viability of the microencapsulated and free probiotic bacteria is presented in Figure 4. The number of probiotics declined significantly as the incubation time increased. The rate of decrease was significantly greater in the free cells (P < 0.05). In the case of free *L.casei*, the cell number was reduced to 7.1×10^3^ CFU/mL after 120 min (Figure 4). The most susceptible cell to intestinal juice was free *B. bifidum *which was reduced to 2.3×10^3^ CFU/mL after 120 min of incubation, whereas in chitosan coated samples, the cell number reduction was about 3.51 and 4.65 log, for *L. casei *and *B. bifidum*, respectively (Figure 4). Our result indicated significantly that alginate-gelatinize starch microcapsules with chitosan coating were most effective in protecting probiotic bacteria from simulated intestinal juice (P < 0.05). This is in good agreement with the results of Krasaekoopt *et al*. (13) in 2004 who indicated that the survival of probiotic bacteria was highly enhanced in gastro-intestinal conditions when encapsulated with alginate-chitosan or poly-L-lysine. Many scientists reported that chitosan coating provides the best protection in bile salt solution since an ion-exchange reaction occurred when the microcapsules absorbed the bile salt (9, 13, 15, 16, 21) therefore the permeability of bile salt into the microcapsules may be restricted. Koo *et al*. (16) in 2001 and Chávarri *et al. *(9) in 2010 reported that *Lactobacillus casei *and *Lactobacillus gasseri *microencapsulated in chitosan-coated alginate beads had higher viability than in microcapsules without chitosan coating in bile salt solution. Sultana *et al*. (25) in 2000 reported that alginate encapsulated *L. acidophilus *and *L. casei *decreased by two log cycles compared to the initial cell count in 1% and 2% bile salt solutions. The protective effect of high amylose maize starch on the bile acid tolerance was measured by Wang *et al*. (35) in 1999. They found that amylomaize promotes the survivability of probiotics by adhesion to starch granules at 0.05% bile acid concentration. The filler material used in preparing microencapsulated probiotic cultures was gelatinized starch. Because of its cross-linked structure, it swells and hence absorbs water. This swollen and gelatinized starch, therefore, contributes to increase an integrated structure and firmness (11, 12, 36, 37). The aim of starch gelatinization and combining with chitosan coating is to improve the structure in order to enhance and extend the industrial applicability. Different studies have shown that calcium alginate microcapsules are better protected in the presence of prebiotics (2, 6, 7, 25, 28, 34). In our study inulin is approved for use in the microencapsulation and it has a positive effect on human health (6, 7). The combination of calcium alginate with prebiotics such as inulin not only improve the viability of probiotics but facilitates formation of an integrated structures of capsules (6, 7, 25, 38, 39). It is important to note that the simulated gastro-intestinal condition (along with pepsin and pancreatin) reported in the literature differs widely in other studies. Most authors prepared simulated gastro-intestinal juice with different concentrations of bile salts and hydrochloric acid and adjusted pH to the desired value without adding pepsin and pancreatin, but seldom with the addition of pancreatin or pepsin to these solutions (9, 27, 29).

## Conclusion

Microencapsulation of *L. casei *and *B. bifidum *in calcium alginate-gelatinized starch with chitosan coating resulted in better survival of cells after simulated gastro-intestinal condition (along with pepsin and pancreatin), as compared to free cells. Therefore the applied approach in this study might prove beneficial for the delivery of probiotic cultures to the simulated human gastro-intestinal tract. Of the two types of microcapsules in this research, chitosan coating provided the best protection of cells and there was no survival of free *B. bifidum *in the presence of gastric juice due to its low acid resistance. The microcapsules used in the present study are micron size and if added to the functional food can deliver a soft texture to the final product.

Future research ought to turn to equipment that can be applicable in the large-scale commercial operations that are commonly employed in food and drug processing industry and related researches are required to assess the efficacy of microencapsulation in the gastro-intestinal tract using animal models.

## References

[1] Food and Agriculture Organization of the United Nations, World Health Organization Expert Consultation (2001). Evaluation of health and nutritional properties of powder milk and live lactic acid bacteria.

[2] Homayouni A, Azizi A, Ehsani MR, Yarmand MS, Razavi SH (2008). Effect of microencapsulation and resistant starch on the probiotic survival and sensory properties of synbiotic ice cream. Food Chem.

[3] Anal AK, Singh H (2007). Recent advances in microencapsulation of probiotics for industrial applications and targeted delivery. Trends Food Sci. Technol.

[4] Aragon-Alegro LC, Alarcon Alegro JH, Roberta Cardarelli H, Chih Chiu M, Isay Saad SM (2007). Potentially probiotic and synbiotic chocolate mousse LWT- Food Sci.. Technol.

[5] Shah NP (2007). Functional cultures and health benefits. Int. Dairy J.

[6] Capela P, Hay TKC, Shah NP (2006). Effect of cryoprotectants, prebiotics and microencapsulation on survival of probiotic organisms in yoghurt and freeze-dried yoghurt. Food Res. Int.

[7] Nazzaro F, Fratianni F, Coppola R, Sada A, Orlando P (2009). Fermentative ability of alginate-prebiotic encapsulated Lactobacillus acidophilus and survival under simulated gastrointestinal conditions. J. Funct. Foods.

[8] Gibson GR (1999). Dietary modulation of the human gut microflora using the prebiotics oligofructose and inulin. J. Nutr.

[9] Chávarri M, Marañón I, Ares R, Ibáñez FC, Marzo F, Villarán MdC (2010). Microencapsulation of a probiotic and prebiotic in alginate-chitosan capsules improves survival in simulated gastro-intestinal conditions. Int. J. Food Microbiol.

[10] Mokarram RR, Mortazavi SA, Najafi MBH, Shahidi F (2009). The influence of multi stage alginate coating on survivability of potential probiotic bacteria in simulated gastric and intestinal juice. Food Res. Int.

[11] Donthidi AR, Tester RF, Aidoo KE (2010). Effect of lecithin and starch on alginate-encapsulated probiotic bacteria. J. Microencapsul.

[12] Mounsey JS, O’Riordan ED (2008). Influence of pre-gelatinized maize starch on the rheology, microstructure and processing of imitation cheese. J. Food Eng.

[13] Krasaekoopt W, Bhandari B, Deeth H (2004). The influence of coating materials on some properties of alginate beads and survivability of microencapsulated probiotic bacteria. Int. Dairy J.

[14] Shu XZ, Zhu KJ (2002). The release behavior of brilliant blue from calcium–alginate gel beads coated by chitosan: the preparation method effect. Eur. J. Pharm. Biopharm.

[15] Lee JS, Cha DS, Park HJ (2004). Survival of Freeze-Dried Lactobacillus bulgaricus KFRI 673 in Chitosan-Coated Calcium Alginate Microparticles. J. Agr. Food Chem.

[16] Koo S, Cho Y, Huh C, Baek Y, Park J (2001). Improvement of the stability of Lactobacillus casei YIT 9018 by microencapsulation using alginate and chitosan. J. Microbiol. Biotechn.

[17] Gupta KC, Ravi Kumar MNV (2000). An Overview on Chitin and Chitosan Applications with an Emphasis on Controlled Drug Release Formulations. J. Macromol. Sci. Polym. Rev.

[18] Fahimdanesh M, Mohammadi N, Ahari H, Zanjani MAK, Hargalani FZ, Behrouznasab K (2012). Effect of microencapsulation plus resistant starch on survival of Lactobacillus casei and Bifidobacterium bifidum in mayonnaise sauce. Afr. J. Microbiol. Res.

[19] Ko JA, Park HJ, Hwang SJ, Park JB, Lee JS (2002). Preparation and characterization of chitosan microparticles intended for controlled drug delivery. Int. J. Pharm.

[20] Mortazavi SA, Rezaei Mokarram A (2004). Preparation and evaluation of diphtheria toxoid-containing microspheres. Iran. J. Pharm. Res.

[21] Murata Y, Toniwa S, Miyamoto E, Kawashima S (1999). Preparation of alginate gel beads containing chitosan salt and their function. Int. J. Pharm.

[22] Simonoska Crcarevska M, Glavas Dodov M, Goracinova K (2008). Chitosan coated Ca–alginate microparticles loaded with budesonide for delivery to the inflamed colonic mucosa. Eur. J. Pharm. Biopharm.

[23] Ribeiro AJ, Silva C, Ferreira D, Veiga F (2005). Chitosan-reinforced alginate microspheres obtained through the emulsification/internal gelation technique. Eur. J. Pharm. Sci.

[24] Allan-Wojtas P, Truelstrup Hansen L, Paulson AT (2008). Microstructural studies of probiotic bacteria-loaded alginate microcapsules using standard electron microscopy techniques and anhydrous fixation. LWT- Food Sci. Technol.

[25] Sultana K, Godward G, Reynolds N, Arumugaswamy R, Peiris P, Kailasapathy K (2000). Encapsulation of probiotic bacteria with alginate–starch and evaluation of survival in simulated gastrointestinal conditions and in yoghurt. Int. J. Food Microbiol.

[26] Krasaekoopt W, Bhandari B, Deeth HC (2006). Survival of probiotics encapsulated in chitosan-coated alginate beads in yoghurt from UHT- and conventionally treated milk during storage. LWT- Food Sci. Technol.

[27] Brinques GB, Ayub MAZ (2011). Effect of microencapsulation on survival of Lactobacillus plantarum in simulated gastrointestinal conditions, refrigeration, and yogurt. J. Food Eng.

[28] Zanjani MAK, Tarzi BG, Sharifan A, Mohammadi N, Bakhoda H, Madanipour MM (2012). Microencapsulation of Lactobacillus casei with calcium alginate-resistant starch and evaluation of survival and sensory properties in cream-filled cake. Afr. J. Microbiol. Res.

[29] Michida H, Tamalampudi S, Pandiella SS, Webb C, Fukuda H, Kondo A (2006). Effect of cereal extracts and cereal fiber on viability of Lactobacillus plantarum under gastrointestinal tract conditions. Biochem. Eng. J.

[30] Hansen LT, Allan-Wojtas PM, Jin YL, Paulson AT (2002). Survival of Ca-alginate microencapsulated Bifidobacterium spp in milk and simulated gastrointestinal conditions.. Food Microbiol.

[31] Yu WK, Yim TB, Lee KY, Heo TR (2001). Effect of skim milk-alginate beads on survival rate of bifidobacteria. Biotechnol. Bioprocess Eng.

[32] Woo C, Lee K, Heo T (1999). Improvement of Bifidobacterium longum stability using cell-entrapment technique. J. Microbiol. Biotechnol.

[33] Lee K, Kim J, Lee Y, Choi E, Shin D, Heo T (2001). Estimating the viability of Bifidobacterium longum in Ca-alginate beads against simulated gastroenteric juices. J. Microbiol. Biotechnol.

[34] Chen KN, Chen MJ, Liu JR, Lin CW, Chiu HY (2005). Optimization of incorporated prebiotics as coating materials for probiotic microencapsulation. J. Food Sci.

[35] Wang X, Brown IL, Evans AJ, Conway PL (1999). The protective effects of high amylose maize (amylomaize) starch granules on the survival of Bifidobacterium spp in the mouse intestinal tract.. J. Appl. Microbiol.

[36] Mohammadi N, Ahari H, Fahimdanesh M, Zanjani MAK, Anvar A, Shokri E (2012). Survival of alginate-prebiotic microencapsulated Lactobacillus acidophilus in mayonnaise sauce. Iran. J. Vet. Med.

[37] Slaughter SL, Ellis PR, Butterworth PJ (2001). An investigation of the action of porcine pancreatic alpha-amylase on native and gelatinized starches. Biochim. Biophys. Acta.

[38] Moghadam Ariaee F, Tafaghodi M (2012). Mucosal adjuvant potential of Quillaja saponins and cross-linked dextran microspheres, co-administered with liposomes encapsulated with tetanus toxoid. Iran. J. Pharm. Res.

[39] Narra K, Dhanalekshmi U, Rangaraj G, Raja D, Kumar CS, Reddy PN, Mandal AB (2012). Effect of formulation variables on rifampicin loaded alginate beads. Iran. J. Pharm. Res.

